# A rare cause of persistent leukocytosis with massive splenomegaly: Myeloid neoplasm with BCR-PDGFRA rearrangement—Case report and literature review

**DOI:** 10.1097/MD.0000000000029179

**Published:** 2022-06-17

**Authors:** Lu Gao, Yan Xu, Lan-chun Weng, Zu-guo Tian

**Affiliations:** a Department of Hematology, Affiliated Hospital of Zunyi Medical University, Zunyi, Guizhou Province, China; b Nursing College, Zunyi Medical University School of Medicine and Technology, Zunyi, Guizhou Province, China.

**Keywords:** BCR-PDGFRA rearrangement, case report, CML, massive splenomegaly, persistent leucocytosis

## Abstract

**Rationale:**

Persistent leukocytosis with megalosplenia is a common manifestation among patients with myeloproliferative neoplasm (MPN), especially for chronic myeloid leukemia (CML) patients. Here, we report a rare case of myeloid neoplasm with BCR-PDGFRA rearrangement characterized by obvious elevation of leukocyte count and megalosplenia.

**Patient concerns:**

A 32-year-old man presented with persistent leukocytosis and megalosplenia.

**Diagnosis:**

This patient was characterized by increased leukocyte count and megalosplenia, and was clinically diagnosed as CML. However, the BCR/ABL fusion gene of the patient was negative, which did not support CML. Moreover, the results of the karyotype showed 46, XY, t(4;22)(q12;q11) and RT-PCR + Sanger detection showed positive PDGFA/BCR. Accordingly, the diagnosis of myeloid neoplasm with BCR-PDGFA rearrangement was confirmed.

**Interventions:**

This patient was initially received imatinib (400 mg) orally once a day, and the dosage was adjusted to 100 mg owing to suffering from grade IV bone marrow suppression.

**Outcomes:**

Hematological remission was achieved after 2 weeks, the best treatment response was achieved after 3 months, and the main molecular biological response was achieved after 12 months.

**Lesson:**

This case suggests that rare PDGFA fusion genes screening for patients comorbid with leukocytosis and megalosplenia is necessary to avoid misdiagnosis. Unlike other rearrangements of PDGFRA, the clinical manifestations of BCR-PDGFRA rearrangement are resembling CML without eosinophilia increase.

## 1 Introduction

Persistent leukocytosis with megalosplenia without any underlying infectious or inflammatory cause is a common manifestation among patients with myeloproliferative neoplasm (MPN), especially chronic myeloid leukemia (CML).^[1–3]^ We report a rare case of myeloid neoplasm with BCR-PDGFRA rearrangement characterized by marked elevation of leukocyte count and megalosplenia, which might improve the focus on this group of diseases and potentially reduce missed diagnoses or misdiagnoses.

## 2 Case presentation

**Figure 1:**
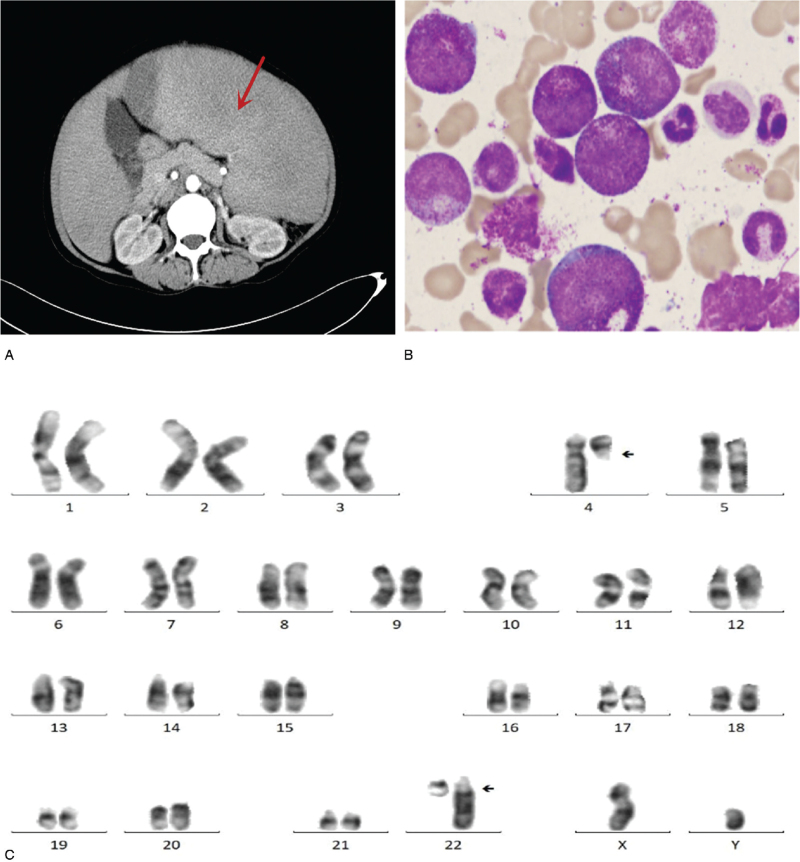
(A) Contrast-enhanced CT scan of the abdomen showing giant spleen. (B) Bone marrow smears refers typical chronic myeloid leukemia morphology (10 × 100). (C) Karyotype 46, XY, t(4;22)(q12;q11) [20].

A 32-year-old man had a chief complaint of persistent abdominal distension for 3 weeks. Physical examination revealed a giant spleen with a hard and smooth texture. Laboratory results revealed: leukocyte count (white blood cell—WBC) 221 × 10^9^/L (normal range 4–10 × 10^9^/L), with normal eosinophils in leukocyte classification, red blood cells count (RBC) 3.34 × 10^12^/L (normal range 3.5–5.5 × 10^12^/L), hemoglobin (HB) 112 g/L (normal range 110–150 g/L), platelet count (PLT) 101 × 10^9^/L (normal range 100–300 × 10^9^/L). Contrast-enhanced CT scan of the abdomen suggested megalosplenia (Fig. 1A).

**Figure 2:**
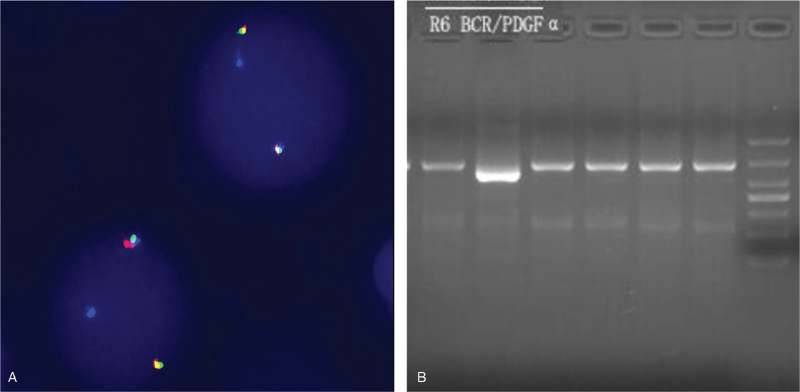
(A) Fish (PDGFRA) results showed that the typical 98% fusion signal [392/400]. (B) BCR/PDGFA positive was detected by using RT-PCR + Sanger.

The morphology of bone marrow showed that nucleated cells proliferated extremely actively (granulocyte:red = 65:1), with abnormal proliferation of granulocytes, significantly increased proportion of neutral lobular nuclei (43%), eosinophils (1.5%), and cytochemical staining showed NAP score of 2 points. The morphology of bone marrow results suggested CML (Fig. 1B). However, BCR/ABL fusion gene (p210/p190/p230), JAK2 gene V617F mutation, calr gene exon 9 mutation, and MPL gene w515L/K mutation were negative in polymerase chain reaction (PCR) detection of bone marrow cells. Fortunately, chromosome abnormalities were found, and G-banding showed 46, XY, t(4;22)(q12;q11) [20] (Fig. 1C). Fluorescence in situ hybridization (FISH) showed that the separation signal of PDGFRA (4q12) was 98% (Fig. 2A). Reverse transcription (RT)-PCR + Sanger showed positive BCR-PDGFA (Fig. 2B). Considering all levels of evidence, the patient was diagnosed as myeloid tumor with BCR-PDGFA rearrangement.

Treatment regimen: orally taking 1 g hydroxyurea 3 times a day (aiming to control WBC to 50 × 10^9^/L), followed by targeted treatment with imatinib mesylate 400 mg orally once a day. However, the patient demonstrated serious signs due to hematological toxicity. We stopped imatinib, according to relevant literature and NCCN guidelines. After recovery of blood cell count, the patient continued to take imatinib 100 mg orally once a day. A complete hematologic remission (CHR) was achieved after imatinib treatment for 2 weeks, a normal karyotype was achieved 3 months later, a complete cytogenetic remission (CCyR) was achieved, with PDGFRA-BCR at 0.043% after 6 months, a major molecular biologic response (MMR) was achieved, and PDGFRA-BCR was 0.012% after 12 months (Table 1). At the submission date, the disease-free survival of the patient was 13 months. Written informed consent was obtained from the patient for publication of this case report. The ethical approval and documentation for this case report was authorized by the Ethical Committee of the Affiliated Hospital of Zunyi Medical University.

**Table 1:** The therapeutic effect of imatinib on Myeloid neoplasm with BCR-PDGFRA rearrangement.

Date	Imatinib (dosage) (mg)	BCR/PDGFA (%)
2020-10-14	100	98
2021-01-12	100	0.1
2021-05-31	100	0.0435
2021-09-18	100	0.012

## 3 Literature review and discussion

It is well known that persistent increased leukocyte count comorbid with enlarged spleen is the most common clinical manifestation of CML. This patient was characterized by increased leukocyte count and megalosplenia, with CML bone marrow, and was clinically diagnosed as CML. However, the BCR/ABL fusion gene of the patient was negative, which did not support CML. As the JAK2 gene V617F mutation, calr gene exon 9 mutation, MPL gene w515l/K mutation were all negative, the MPN diagnosis could be established as well.^[4,5]^ At this time, the case diagnosis was in distress. It needs to be considered that there may be other rare causes of persistent increased leukocyte count and megalosplenia.

Fortunately, the results of the karyotype showed 46, XY, t(4;22)(q12;q11), Fish PDGFRA (4q12) showed 98% separated signal, and RT-PCR + Sanger detection showed positive PDGFA/BCR. Therefore, the diagnosis of myeloid neoplasm with BCR-PDGFA rearrangement was confirmed.

Myeloid/lymphoid neoplasms associated with eosinophilia and rearrangements of PDGFRA, PDGFRb, or FGFR1 or PCM1-JAK are three particularly rare diseases.^[6]^ This case warned us that it is necessary to perform test for rare fusion genes associated with PDGFA for patients with markedly increased leukocytes count comorbid with megalosplenia to avoid missed diagnoses.

The most common gene fusions of myeloid neoplasms associated with PDGFRA rearrangements are the FIPI-PDGFRA fusions formed by recessive deletion of 4q12 and occasionally other variant fusion gene types, such as KIF5B-PDGFR, CDK5RAP2-PDGFRA,ETV6-PDGFRA,STRN-PDGFRA, TNKS2-PDGFRA, and BCR-PDGFRA.^[7–9]^ Such disorders, mainly characterized by multisystem damage caused by eosinophilic infiltration in clinical setting.

To the best of our knowledge, only 10 patients with t(4; 22) (q22; q11)/BCR-PDGFRA cases has been reported around the worldwide in published literature^[7,9–14]^ (Table 2), including 9 males and 2 females, with a mean age of 39 years, a minimum age of 3 years, and a maximum of 57 years. The dominant clinical features are both leukocyte count and splenomegaly, without evidence of eosinophilia. The diagnoses were atypical CML (n = 2), CML like MPD with extramedullary T-lymphoid blast crisis (n = 1), Pre-B cell ALL (n = 1), CEA (n = 2), mixed phenotypic acute leukemia (B/myeloid) (n = 1), T-lymphoblastic leukemia/lymphoma (T-ALL) (n = 1), B-ALL (n = 1), and MPN (n = 2).

**Table 2:** Clinical features and treatment outcomes of cases with BCR-PDGFRA rearrangements implicated in the literature.

Case no.	Sex/age	Physical examination	Hemogram	Karyotype	BCR-PDGFRA fusion transcripts	Diagnosis	Treatment regimens	Follow-up	Ref
1	M/37	Splenomegaly	Leukocytosis(WBC57 × 10^9^/) Eosinophils (5%)	46;XY;t(4;14)(q12;q24)	BCR exon 7 followed by 24 bp of the beginning of BCR intron 7, followed by PDGFRA sequence, exon 12	Atypical CML	Matched allotransplant	Survival	Baxter^[9]^
2	M/3	Enlarged tonsils;lymphadenopathyliver and spleen enlargement	Leukocytosis(WBC101 × 10^9^/L)Eosinophils (22%)	46;XY;t(4;14)(q12;q24)	BCR exon 12 followed by a 12 bp insert followed by PDGFRA sequence, exon 12	CML-like myeloproliferative disorder with extramedullaryT-lymphoid blast crisis	Auto-HSCT PRAllo-HSCT (MSD)	Died on +50 d	Baxter^[9]^
3	M/47	Diffuse ecchymosisMultiple lymphadenopathiesHepatosplenomegaly	Leukocytosis(WBC139 × 10^9^/L)Eosinophils (4%)	45,Y, t(3;12)(p23;q14),del(9)(p21), t(4;22)(q12;q11),der(9)ins(9;?)(q12;?)	BCR exon 1 with PDGFRA exon 13	Pre-B cell ALL	(BCR-PDGFRA 95% pretreatment) induction (VDCLP) CR 5 wk laterBCR-PDGFRA 100% consolidation (HD-MTX + Lasp) BCR-PDGFRA 70% Glivec 400 mg/d CHR within 6 wk DBCR-PDGFRA 15% PCyR within 4 wk maintained imatinib	Survival	Trempat^[11]^
4	M/57	AplenomegalyLymphadenopathy	Leukocytosis(WBC51 × 10^9^/L)Eosinophils (13%)	46;XY;t(4;14)(q12;q24)	BCR intron 17 (position143,925) and PDGFRA exon 12 (position 1836)	Atypical CML	Imatinib 100 mg/d Hematologic response within 1 mo; A 7 mo follow up normal blood counts	Survival	Safley^[10]^
5	M/37	N/A	N/A	46,XX, t(4;22)(q12;q11)	N/A	CEL	N/A	N/A	Philipp^[14]^
6	M/41	N/A	N/A	46,XX, t(4;22)(q12;q11)	N/A	CEL	N/A	N/A	Philipp^[11]^
7	M/45	Cervical lymphadenopathy	Leukocytosis(WBC59 × 10^9^/L)	46,XX, t(4;22)(q12;q11.2)	N/A	Mixed phenotypic acute leukemia	Induction (IA + imatinib) MCR within 28 dAllo-HSCT (WM-URD)	Survival	Wang^[7]^
8	M/56	Marked splenomegalyLymphadenopathy	Leukocytosis(WBC26.3 ×10^9^/L)Eosinophils (2%)	46,XY, t(4;22)(q12;q11.2)	N/A	T-ALL	Induction (protocol-10102) CR within 3 mo after the diagnosisIntensive induction and consolidationRegimens, treatment was followed bymaintenance therapy for a total of 2 yr	Remained in CR for 4 yr	Yigit^[12]^
9	M/37	N/A	Leukocytosis(WBC52 × 10^9^/L)Eosinophils (1%)	46,XY, t(4;22)(q12;q11)	N/A	Myeloproliferative neoplasm	Started on Imatinib CHR within 1 mo	Survival	Manish^[8]^
10	M/77	Lympoadenopathy	Leukocytosis(WBC2.4 × 10^9^/L)	39,XY,-3,-7,-13,-14,-15,16,1[3]/78,idemx2,10,+13[9]/74,idemx2, t(2;5)(p21;p14),4[3]/46,XY[5]	N/A	B-ALL therapy related myeloid neoplasm	Induction Rituxan plus HyperCVAD AND POMP plus Rituxan CR MRD negative13 mo later relapse	Died	Zhou^[13]^
11	M/32	Splenomegaly	Leukocytosis(WBC221 × 109/L)Eosinophils (1.5%)	46,XY, t(4;22)(q12;q11)	BCR exon 15 with PDGFRA exon12	Myeloid neoplasm with PDGFRA-BCR rearrangement	Imatinib 100 mg CHR within 1 mo, MMR within 12 mo	Survival	Present case

ALL = acute lymphoblastic leukemia, allo-HSCT = allogeneic hematopoietic stem cell transplantation, CEL = chronic eosinophilic leukemia, CHOP = cyclophosphamide, doxorubicin, vincristine, prednisone, CHR = complete hematological remission, CR = complete remission, CT = chemotherapy, del = deletion, dup = duplication, F = female, IA = idarubicin, cytarabine, M = male, MCR = major cytogenetic remission, MMR = major molecular biological remission, MRD = minimal residual disease, MSD = matched-sibling donor, NA = not available, PCy = partial cytogenetic remission, PR = partial remission, SCT = stem cell transplantation, T-ALL = T-cell ALL, VDCLP = vincristine, daunorubicin, cyclophosphamide, l-asparaginase, prednisone, WBC = white blood count.

BCR-PDGFRA rearrangement, with a clinical presentation different from that of other rearrangements of PDGFRA, without eosinophilia increase and with a clinical presentation resembling CML. Of these cases that have been reported, 1 case was treated with hydroxyurea with poor prognosis and disease progression. Three patients were treated with HSCT (one with autologous HSCT and two with allogeneic HSCT). There were 3 patients who choosing imatinib treatment, 2 patients receiving imatinib at a dose of 100 mg orally once a day, and 1 patient receiving imatinib at a dose of 400 mg orally but having severe hematologic toxicity, which was changed to 100 mg. Three patients all achieved hematologic remission within 2 weeks and survived during follow-up.

Myeloid neoplasms with BCR-PDGFRA rearrangements aberrantly express tyrosine kinases and are sensitive to treatment with tyrosine kinase inhibitors, with several fold greater sensitivity than BCR/ABL related diseases.^[15]^ Therefore, a tyrosine kinase inhibitor, imatinib, is the first line therapeutic agent for the treatment of this category of diseases.^[3]^ This patient was initially received imatinib (400 mg) orally once a day, but the patient suffered grade IV bone marrow suppression after 1 week. After stopping imatinib and supporting treatment with cell growth factor, the blood cell count returned to normal. The dose was changed to 100 mg orally once a day. Hematological remission was achieved after 2 weeks, the best treatment response was achieved after 3 months, and the main molecular biological response was achieved after 12 months.

We reported the case of myeloid neoplasm with BCR-PDGFRA rearrangement, who rapidly achieved hematologic and genetic remission after treatment with imatinib, and achieved a major molecular remission after 12 months of treatment. It remains to be seen whether the prognosis of the patient is as good as that of CML.

## Acknowledgment

The author thanks the patients for his approval to publication.

## Author contributions

**Investigation:** Yan Xu, Lan-chun Weng.

**Project administration:** Zu-guo Tian.

**Writing – original draft:** Lu Gao.

**Writing – review & editing:** Lu Gao.
